# Supplementary material for: Chromothripsis during telomere crisis is independent of NHEJ, and consistent with a replicative origin
Source: Genome Res. 2019 May;29(5):737–49. doi: 10.1101/gr.240705.118 (PMC6499312; doi:10.1101/gr.240705.118)
Supplement: Supplemental Material [file supp_gr.240705.118_Supplemental_file_1.zip › contigs/annotated_contigs/DB53/contig.3.DB53_length_677_mean_cov_6.61742983752.docx]

**DB53_length_677_mean_cov_6.61742983752**

```
TCTTTAGCTTCAATTGCTTGCGATTTTTCTTTAATGGTACAATTAGGTTTTCTGTGAGTGCATGTGGTTAAAAAATAAAAACTGAGTCT
 >chr8:81444122-81444512 - E=7e-214
CTTTCTTTAGCATATATAAATGCTACATTCTGCCTTTGCAATTACTCCATTAGTCATCTCCAACAATGAATTATGGTGATAAAGGAGGT

ACTATTTTTTAAAATATGCCACTAGAAAGGACATTAATTGTAGTATAATATACTGGAAACATTCTCCAATCTGATTTTTCAAGTTATTT

ACATCATAAATGCAGAAAACAAACTTCTACAGTGGTAAATGCATATTTACCACTACAGCTAATGCATATTTATCAATTTCCTGCTTGCA

AAAAATACTGCATGTCTGTATCATTTGCACT|TAG|AAGAAAACCTAGGCATTACCATTCAGGACATAGTCATGGGCAAGGACTTCATG
                                >chr8:76241353-76241621 + E=2e-149 p=1e-03
TCCGAAACACCAAAAGCAATGGCAACAAAAGCCAAAATTGACAAATGGGATCTAACTAAACTAAAGAGCCTCTGCACAGCAAAAGAAAC

TACCATCAGAGTGAACAGGCAACCTACAAAATGGGAGAAAATTTTCGCAACCTACTCATCTGACAAAGGGCTAATATCCAGAATCTACA

ATGAACTCAAACAAATTTACAAG|AAAAAAACAAA|AAAAAAAAAAAAAAAGTGGGTG                        >chr4:17668552-17668585 - E=4e-0
```
